# Neutrophil-to-Lymphocyte Ratio as an Early Predictor of Complication and Mortality Outcomes in Individuals With Acute Pancreatitis at a UK District General Hospital: A Retrospective Analysis

**DOI:** 10.7759/cureus.29782

**Published:** 2022-09-30

**Authors:** Sattam A Halaseh, Marcos Kostalas, Charles Kopec, Ahmad A Toubasi, Rola Salem

**Affiliations:** 1 General and Colorectal Surgery, Torbay and South Devon NHS Foundation Trust, Torbay Hospital, Torquay, GBR; 2 Upper Gastrointestinal Surgery, Torbay and South Devon NHS Foundation Trust, Torbay Hospital, Torquay, GBR; 3 Medicine, Faculty of Medicine, University of Jordan, Amman, JOR

**Keywords:** neutrophil-to-lymphocyte ratio, complication, mortality, pancreatitis, surgical acute abdomen

## Abstract

Aim

The purpose of this study was to evaluate the predictive usefulness of the neutrophil-to-lymphocyte ratio (NLR) in patients with acute pancreatitis (AP) and establish a threshold for the prediction of poor outcomes.

Methods

For this investigation, we looked back at all cases of acute pancreatitis treated at Torbay Hospital in Torquay, UK, between January 1st, 2019, and December 31st, 2020. Those who were found to have chronic pancreatitis or whose baseline laboratory values could not be obtained were not included. Each patient’s entire hospital stay was analyzed, including up to 72 hours of medical and laboratory data.

Results

According to the Glasgow Coma Scale scoring system, 28 of the 314 included patients had severe acute pancreatitis, and 81 patients had pancreatitis with complications. Those with complications had a substantially higher NLR on day 1 (9.43 ± 7.57) than patients who recovered without complications (7.37 ± 5.88) (P-value = 0.028). The NLR on day 0 (>18.71) exhibited a sensitivity of 80%, a specificity of 90.2%, and an accuracy of 83.9% in forecasting the death of patients with pancreatitis.

Conclusion

Elevated baseline NLR corresponds with pancreatitis with complications and can predict mortality.

## Introduction

Acute pancreatitis (AP) is a frequent condition with a prevalence of 50 per 100,000 people per year in the United Kingdom, which is on the rise [1]. Pancreatitis is an inflammation of the pancreas and a frequent gastrointestinal hospitalization cause. Pancreatitis is characterized by severe upper abdominal discomfort, nausea, and vomiting. The diagnosis of pancreatitis requires the presence of two out of the following three diagnostic criteria: sudden upper abdominal pain, serum amylase and/or lipase level rise >3 times the upper normal limits, and distinctive abdominal imaging abnormalities [2]. In comparison to mild pancreatitis, which has a death rate of 1.5%, severe pancreatitis has a mortality rate of up to 30% [3]. In severe cases of acute pancreatitis, diagnostic and therapeutic interventions have led to a decrease in mortality [4]. Consequently, early detection of the severity of acute pancreatitis is essential and will aid in the identification of individuals who require particular therapies or critical care support.

Numerous grading methods have been described, such as the Acute Physiology and Chronic Health Evaluation II (APACHE II), Ranson’s criteria, Glasgow Coma Scale score, Balthazar score based on clinical and biochemical results, and computed tomography (CT) findings [5,6]. However, none of them are sensitive or detailed enough, and there is no agreement on which grading system should be employed. Furthermore, Ranson’s criteria require 48 hours to complete, limiting their potential to forecast acute pancreatitis severity at the time of first diagnosis and treatment.

White blood cell (WBC) counts and C-reactive protein (CRP) values are nonspecific indicators of systemic inflammation that may be detected by regular hematological serum testing. In addition, the WBC count is associated with poor prognosis as a component of Ranson’s criteria, Glasgow Coma Scale score, and Acute Physiology and Chronic Health Evaluation II (APACHE II), which are acute pancreatitis prognostic grading systems [7-9]. Nonetheless, the total WBC count can change dependent on a variety of physiological and pathological factors, such as hydration state, stress, and pregnancy, as well as how the blood samples are handled [8]. The neutrophil-to-lymphocyte ratio (NLR) has been recognized as a more accurate predictor of unfavorable outcomes in numerous benign and malignant disorders than the WBC count in esophageal cancer, colorectal cancer, and coronary heart disease [10-12]. Neutrophils and lymphocytes are stronger indicators of the immunological response than the total WBC count [11,12]. Specifically, investigations have revealed the link between systemic lymphocytopenia and acute pancreatitis severity [13,14]. In addition, one study demonstrated that the NLR predicts the severity of acute pancreatitis more accurately than the total WBC count [8].

In addition to demonstrating the value of the NLR in predicting death in patients with acute pancreatitis, the purpose of this study was to establish the NLR’s ability to predict the occurrence of complications in the early stages of the disease. In addition, the appropriate NLR cutoff value for predicting bad outcomes in individuals with acute pancreatitis was investigated.

## Materials and methods

**Figure 1:**
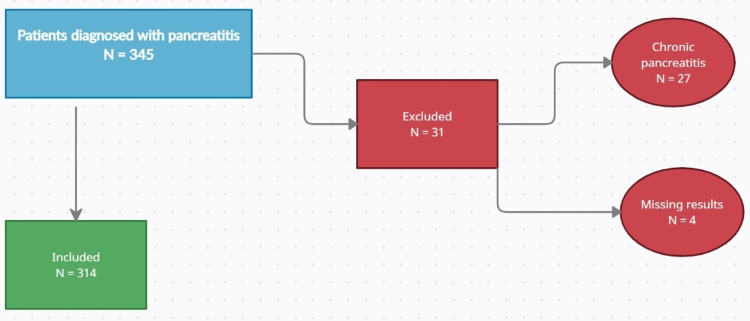
Patient selection flowchart.

This study was designed as a single-center, retrospective analysis of all acute pancreatitis patients treated at Torbay Hospital in Torquay, United Kingdom, between January 1st, 2019, and December 31st, 2020. Throughout the hospitalization of each patient, medical and laboratory data for up to 72 hours were evaluated. Patients whose baseline laboratory results were unavailable or identified with chronic pancreatitis were excluded from the study. A total of 31 patients were excluded from this analysis: 27 had chronic pancreatitis, and four had missing laboratory results (Figure 1).

Definition

Acute pancreatitis was diagnosed in patients who met at least two of the following criteria: abdominal discomfort coherent with acute pancreatitis (acute onset of a prolonged, severe, epigastric pain that frequently radiates to the back), serum amylase level at least three times the upper limit of the standard value, and the distinctive manifestation of acute pancreatitis on contrast-enhanced computed tomography (CECT) [7]. Mild pancreatitis, moderate pancreatitis, and severe pancreatitis were classified based on the Glasgow Coma Scale score, with a total score of 3 and above indicating severe acute pancreatitis [15].

Analysis

The patients’ data were entered into Microsoft Office Excel 2019 (Microsoft Corp., Redmond, WA, USA) and then imported into Statistical Package for the Social Sciences (SPSS) software version 25 (IBM Corp., Armonk, NY, USA), which was used to conduct the analysis. Categorical variables were presented as counts and percentages, while continuous variables were analyzed using means and standard deviations. T-test and chi-square test were used as appropriate to identify the variables associated with pancreatitis outcomes. A receiver operating characteristic (ROC) curve analysis was done to investigate the predictive value of the NLR in pancreatitis. All variables with a P-value of <0.05 in the tests were considered statistically significant.

## Results

Characteristics of included patients

The total number of included patients with pancreatitis was 314. The female sex represented 51.3% of the study population, while the rest were male. The mean ± standard deviation age of the patients was 56.33 ± 17.41. The most common cause of pancreatitis among the included patients was gallbladder stones (39.5%), followed by alcohol (16.6%). The majority of the patients received supportive treatment in the hospital (78.6%). The mean ± standard deviation white blood cell count was 11.86E3 ± 5.10E3. Additionally, the mean ± standard deviation C-reactive protein (CRP) was 39.81 ± 71.42, while the mean ± standard deviation peak CRP was 123.29 ± 134.55. The mean ± standard deviation neutrophil and lymphocyte counts were 10.14E3 ± 4.64E3 and 1.468 ± 0.85E3, respectively. Table 1 describes the characteristics of the included patients.

**Table 1:** Characteristics of included patients. n: number; ERCP: endoscopic retrograde cholangiopancreatography

Variable	Response	Frequency (n = 314)	Percentage (%)
Sex	Male	161	48.7
	Female	153	51.3
Cause of pancreatitis	Alcohol	76	24.2
	Stone	156	49.68
	Drug induced	5	1.5
	Idiopathic	40	12.7
	Hyperlipidemia	2	0.6
	Autoimmune	2	0.6
	Iatrogenic	4	1.2
	Unclear etiology	29	9.2
In-hospital treatment	Supportive	246	78.6
	Procedure (ERCP)	15	4.8
	Surgery	52	16.6
Severity according to Glasgow Coma Scale score	Not severe	286	91.1
	Severe	28	8.9
Complications	Self-limited	233	74.2
	Complicated	81	25.8
Outcome	Discharged	309	98.4
	Death	5	1.6

The association of characteristics and laboratory findings with mortality

Regarding patients’ mortality, 1.6% of the included patients died. There was a significant difference in age between recovered patients and deceased ones as the mean ± standard deviation age of deceased patients was 77.60 ± 13.11, while it was 55.99 ± 17.28 for recovered patients (P-value = 0.006). Furthermore, deceased patients had significantly higher CRP levels compared to recovered patients (P-value = 0.005). Similarly, peak CRP levels were significantly higher among deceased patients compared to recovered ones (P-value = 0.048). In addition, there was a significant difference in the NLR on day 0 between the two groups as deceased patients (19.67 ± 8.01) had significantly higher NLR means compared to recovered patients (9.92 ± 10.15) (P-value = 0.033). However, there was no significant difference between the two groups on the NLR on day 1 or 2 (Table 2).

**Table 2:** Association of characteristics and laboratory findings with mortality. PO_2_: partial pressure of oxygen; NLR: neutrophil-to-lymphocyte ratio; day 0: time of admission; day 1: after 24 hours of admission; day 2: after 48 hours of admission

Variable		Death (n = 5)	Discharged (n = 309)	P-value
Gender	Females	2 (40)	151 (48.9)	0.694
	Males	3 (60)	158 (51.1)	
Age (year)		77.60 ± 13.11	55.99 ± 17.28	0.006^*^
Amylase (IU/L)		478.20 ± 517.68	866.69 ± 1055.42	0.413
C-reactive protein (mg/L)		128.20 ± 110.41	38.314 ± 69.91	0.005^*^
PO_2_ (kPa)		4.67 ± 2.81	9.6937 ± 37.62777	0.818
White blood cells (10^9^/L)		11.86E3 ± 5.13E3	11.580E3 ± 3.5096E3	0.904
Calcium (mmol/L)		2.37 ± 0.064	2.39 ± 0.31	0.934
Urea (mmol/L)		6.54 ± 3.89	6.53 ± 8.87	0.997
Lactate dehydrogenase (IU/L)		409.50 ± 74.246	555.67 ± 310.45	0.509
Aspartate transferase (IU/L)		142.00 ± 230.77	130.01 ± 174.391	0.892
Albumin (g/L)		43.60 ± 3.975	43.32 ± 6.56	0.925
Blood glucose (mmol/L)		11.025 ± 7.3418	9.04 ± 5.77	0.496
Peak C-reactive protein (mg/L)		241.00 ± 98.42	121.30 ± 134.32	0.048^*^
NLR day 0		19.67 ± 8.01	9.92 ± 10.15	0.033^*^
NLR day 1		12.80 ± 7.20	7.91 ± 6.47	0.136
NLR day 2		13.81 ± 4.90	7.56 ± 7.21	0.086

The association of characteristics and laboratory findings with pancreatitis severity

The Glasgow Coma Scale scores demonstrated that 8.9% of the included patients had severe pancreatitis. Patients with severe pancreatitis had significantly higher amylase levels compared to patients with non-severe pancreatitis (P-value = 0.002). Moreover, CRP levels were also significantly higher among patients with severe pancreatitis (P-value = 0.020). Furthermore, patients with severe pancreatitis had significantly higher means of white blood cells, urea, lactate dehydrogenase, aspartate transferase, blood glucose, and peak CRP (P-value < 0.05). On the other hand, none of the NLR were significantly different between the two groups (Table 3).

**Table 3:** Association of characteristics and laboratory findings with pancreatitis severity. PO_2_: partial pressure of oxygen; NLR: neutrophil-to-lymphocyte ratio; day 0: time of admission; day 1: after 24 hours of admission; day 2: after 48 hours of admission

Variable		Severe (n = 28)	Non-severe (n = 286)	P-value
Gender	Females	12 (42.9)	141 (49.3)	0.515
	Males	16 (57.1)	145 (50.7)	
Age (year)		57.54 ± 17.39	56.21 ± 17.44	0.702
Amylase (IU/L)		1,432.11 ± 1,421.92	802.29 ± 989.18	0.002^*^
C-reactive protein (mg/L)		69.64 ± 85.78	36.74 ± 69.24	0.020^*^
PO_2_ (kPa)		7.02 ± 7.81	9.98 ± 39.81	0.735
White blood cells (10^9^/L)		16.55E3 ± 5.96E3	11.39E3 ± 4.77E3	0.000^*^
Calcium (mmol/L)		2.31 ± 0.19	2.39 ± 0.32	0.185
Urea (mmol/L)		11.15 ± 8.34	6.07 ± 8.74	0.003^*^
Lactate dehydrogenase (IU/L)		830.00 ± 282.96	512.46 ± 291.73	0.000^*^
Aspartate transferase (IU/L)		241.96 ± 221.44	118.62 ± 165.46	0.000^*^
Albumin (g/L)		41.57 ± 5.53	43.50 ± 6.60	0.136
Blood glucose (mmol/L)		15.04 ± 9.88	8.42 ± 4.76	0.000^*^
Peak C-reactive protein (mg/L)		188.43 ± 130.69	116.61 ± 133.38	0.007^*^
NLR day 0		10.19 ± 10.36	8.91 ± 8.21	0.535
NLR day 1		10.02 ± 7.26	7.82 ± 6.42	0.170
NLR day 2		8.00 ± 6.52	7.66 ± 7.28	0.864

The association of characteristics and laboratory findings with complications

Regarding the occurrence of complications among the included patients, complications occurred in 25.8% of the study population. None of the patients’ characteristics or laboratory findings were significantly associated with the occurrence of patient complications except the NLR. There was a significant difference in the NLR on day 1 between patients who had complications compared to patients who recovered without any complications as patients who had complications had significantly higher NLR on day 1 (9.43 ± 7.57) compared to the other group (7.37 ± 5.88) (P-value = 0.028). Additionally, patients who developed complications had significantly higher NLR on day 2 (9.96 ± 10.46) compared to their counterparts (6.71 ± 5.02) (Table 4).

**Table 4:** Association of characteristics and laboratory findings with complications. PO_2_: partial pressure of oxygen; NLR: neutrophil-to-lymphocyte ratio; day 0: time of admission; day 1: after 24 hours of admission; day 2: after 48 hours of admission

Variable		Complicated (n = 81)	Self-limiting (n = 233)	P-value
Gender	Females	43 (53.1)	110 (47.2)	0.362
	Males	38 (46.9)	123 (52.8)	
Age (year)		54.73 ± 16.98	56.89 ± 17.57	0.337
Amylase (IU/L)		706.25 ± 890.78	916.26 ± 1,098.03	0.123
C-reactive protein (mg/L)		44.15 ± 73.41	38.24 ± 70.79	0.527
PO_2_ (kPa)		5.91 ± 3.01	11.16 ± 44.40	0.410
White blood cells (10^9^/L)		11.63E3 ± 5.59E3	11.94E3 ± 4.93E3	0.640
Calcium (mmol/L)		2.43 ± 0.50	2.37 ± 0.21	0.151
Urea (mmol/L)		7.08 ± 13.11	6.33 ± 6.71	0.512
Lactate dehydrogenase (IU/L)		443.30 ± 168.04	580.05 ± 329.03	0.056
Aspartate transferase (IU/L)		128.56 ± 174.04	130.71 ± 175.39	0.927
Albumin (g/L)		44.37 ± 7.26	42.96 ± 6.22	0.095
Blood glucose (mmol/L)		9.35 ± 6.26	8.96 ± 5.62	0.615
Peak C-reactive protein (mg/L)		140.13 ± 150.86	117.40 ± 128.21	0.200
NLR day 0		10.28 ± 11.18	9.49 ± 6.60	0.546
NLR day 1		9.43 ± 7.57	7.37 ± 5.88	0.028^*^
NLR day 2		9.96 ± 10.46	6.71 ± 5.02	0.003^*^

The predictive value of the NLR in patients with pancreatitis

**Figure 2:**
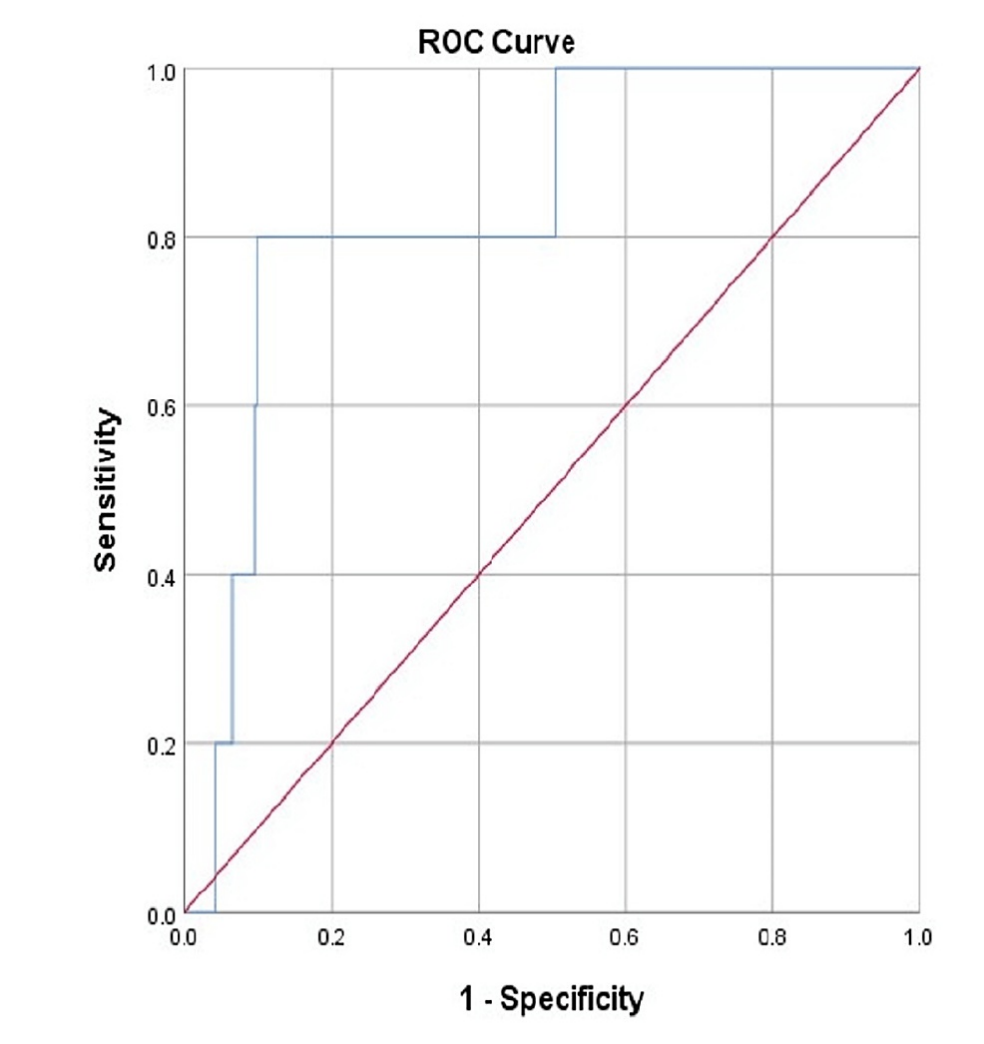
ROC curve representing the NLR on day 0. ROC: receiver operating characteristics

**Figure 3:**
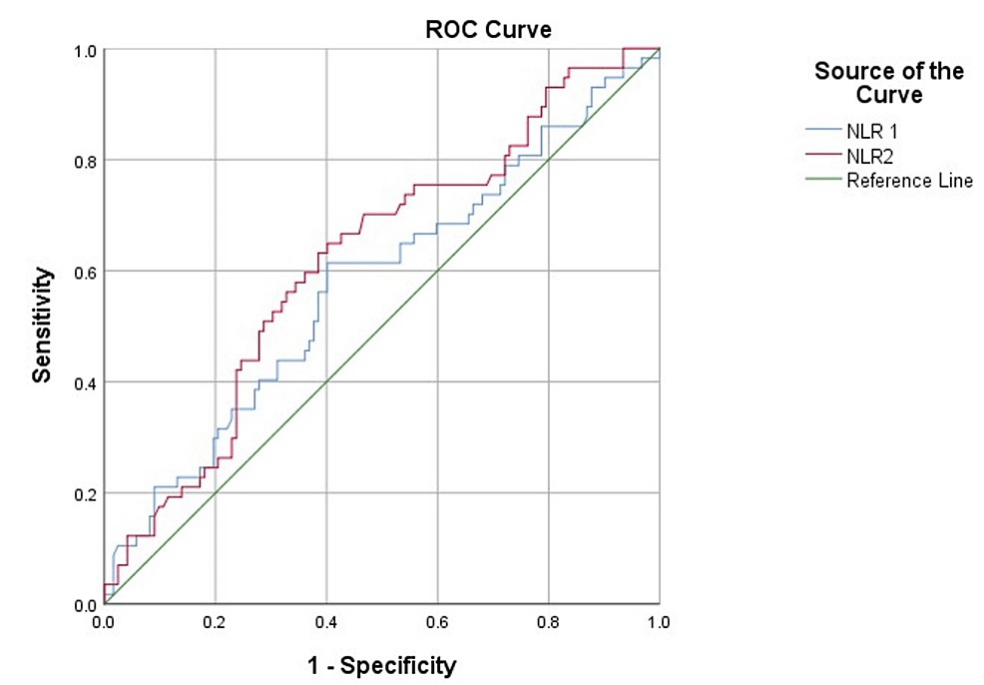
ROC curve representing the NLR on days 1 and 2. ROC: receiver operating characteristics; NLR: neutrophil-to-lymphocyte ratio; NLR1: neutrophil-to-lymphocyte ratio after 24 hours of admission (day 1); NLR2: neutrophil-to-lymphocyte ratio after 48 hours of admission (day 2)

The receiver operating characteristic (ROC) curve showed that the NLR on day 0 was a significant predictor of patient mortality (Figure 2). The NLR on day 0 (>18.71) had a sensitivity of 80%, a specificity of 90.2%, and an accuracy of 83.9% in predicting patients’ mortality. Moreover, the NLR on day 1 and day 2 were significant predictors for the occurrence of complications (Figure 3). The sensitivity, specificity, and accuracy of the NLR on day 1 (>7.70) in predicting patients’ complications were 61.4%, 59.8%, and 57.6%, respectively. In addition, the NLR on day 2 (>6.81) had a sensitivity of 63.2%, a specificity of 61.5%, and an accuracy of 62.1% in predicting patients’ complications (Table 5).

**Table 5:** Sensitivity, specificity, and accuracy of the NLR on day 0 for the prediction of mortality and on days 1 and 2 for the prediction of complications. NLR: neutrophil-to-lymphocyte ratio; day 0: time of admission; day 1: after 24 hours of admission; day 2: after 48 hours of admission

Criteria		Accuracy	Sensitivity	Specificity
Outcome	NLR day 0 (>18.71)	83.9%	80%	90.2%
Complications	NLR day 1 (>7.70)	57.6%	61.4%	59.8%
	NLR day 2 (>6.81)	62.1%	63.2%	61.5%

## Discussion

In the majority of individuals, acute pancreatitis (AP) progresses without problems and necessitates only brief hospitalization. The remaining one-fifth of individuals, however, have a more challenging clinical trajectory. Significant death rates may be associated with long-term critical care admissions, long-term institutionalization, and surgical intervention for these patients [16].

In this study, we assessed the use of the NLR as an early indicator for anticipating the onset of complications and death in patients admitted to our district general hospital with acute pancreatitis. According to these findings, patients with unusually increased NLR levels had a worse prognosis following acute pancreatitis. Our results demonstrate a considerably higher risk of death in patients with an elevated NLR upon admission and highlight the greater risk of requiring intensive care when this marker is raised.

The white blood cell count is commonly utilized as an infection and inflammatory indicator. Some grading systems used to evaluate the outcome of AP, such as the APACHE II and Ranson’s criteria, incorporate the WBC count [17,18]. Although the total WBC count is a component of numerous grading systems for pancreatitis, unlike CRP or blood urea nitrogen, the total WBC count is not evaluated as an independent marker for predicting the outcome of acute pancreatitis [9]. The NLR is derived from the differential count of the previously employed WBC counts. Therefore, more research is unnecessary, and it may be simply used to treat acute pancreatitis. Furthermore, the NLR is a simple, affordable test that is conducted frequently at the first evaluation of patients, is unaffected by the patient’s hydration status, and is easily repeatable. Because neutrophilia and lymphopenia are indicators of inflammatory processes and stress responses, they can more accurately represent outcomes such as necrosis and organ failure [8,9]. In acute pancreatitis, neutrophils stimulate cytokine production pathways of interleukin (IL)-6, IL-8, and proteolytic enzymes such as elastase and free radical formation, hence boosting inflammation and tissue breakdown [8].

Several previous studies, however, have identified the NLR as a marker that represents the prognosis of numerous benign inflammatory or cancerous diseases [11,12,19]. This study indicated that the NLR is raised in individuals with acute pancreatitis and that the NLR may be used to forecast which individuals will develop complications and die. In particular, identifying individuals in the earliest stages of presentation is crucial for improving prognosis [5,20]. Furthermore, there is a requirement for a simple indicator that can forecast the patient’s prognosis within 24 hours of clinical onset [5].

Several prior studies looked into the link between the NLR and the outcome of acute pancreatitis. One study demonstrated that the NLR is preferable over the WBC for predicting intensive care unit admission [8].

In the current study, ROC curve analysis was conducted to predict the likelihood of systemic complications in individuals with AP, and cutoff levels were selected upon admission to the accident and emergency department and at 24 and 48 hours following admission. Regarding the incidence of problems among the enrolled patients, 25.8% of the sample population had complications. Except for the NLR, none of the patient features or laboratory data were substantially linked with patient complications. In our analysis, the NLR cutoff values (>7.70) at 24 hours after admission and the NLR cutoff values (>6.81) at 48 hours after admission acquired for the prediction of systemic complications were close to the average of the NLR cutoff values obtained by previous studies, which demonstrated cutoff values >4.7 and >10.6 [8,9]. This indicates that our research concurs with previously published research. Area under the curve (AUC) values of 0.576 at 24 hours and 0.621 at 48 hours after admission were derived using ROC curve analysis based on NLR values at 24 and 48 hours after admission. Both had a sensitivity and specificity of 0.614 and 0.632, and 0.598 and 0.615, respectively.

In addition, several studies have demonstrated a correlation between mortality and the NLR value in AP. In 2017, in a retrospective analysis, 359 individuals with AP were categorized as either living or deceased. They discovered that the NLR was much greater in the cohort that died compared with the group that lived. In addition, the ROC curve analysis done for the prediction of mortality found an appropriate cutoff value of 16.64 for the NLR on admission, with sensitivity and specificity values of 82.4% and 75.6%, respectively, and an AUC value of 0.80 [21]. In our study, the NLR cutoff value (>18.71) acquired at admission to predict death was highly significant. The AUC of the ROC curve analysis based on NLR levels at admission was estimated to be 0.839, with a sensitivity and specificity of 80% and 90.2%, respectively, which is more significant than the previous study.

Limitations

Our analysis has a few drawbacks. The first is our study’s single-center design. The second limitation is the low number of patients included in the study. It is also possible that the blood test results were influenced by patients’ delayed presentation to the hospital. To examine more precise parameter estimates of the NLR for severe pancreatitis, an additional prospective multicenter trial investigation is required.

## Conclusions

Higher NLR was linked to more complications in individuals hospitalized with acute pancreatitis. Furthermore, it serves as a reliable indicator of their mortality. To identify the potential for complications and mortality in patients who are hospitalized with acute pancreatitis, we propose the use of the NLR as a predictive biomarker.
